# Supplementary material for: Surface Microbiome Profiling of Dental Elevators Using Third‐Generation Sequencing: Implications for Infection Control in Dental Practice
Source: Microbiologyopen. 2025 Dec 8;14(6):e70178. doi: 10.1002/mbo3.70178 (PMC12685768; doi:10.1002/mbo3.70178)
Supplement: Supplementary file 1 — customer_backup. [file MBO3-14-e70178-s001.zip › customer_backup/customer_backup/taxa_summary/krona/groups/treat/M07.Krona.html]

Javascript must be enabled to view this page.

members
magnitude
magnitudeUnassigned

M07.krona

47756

47754

48

0

0

0

0

0

0

0

0

0

0

0

0

0

0

0

48

48

48

48

13

1

2

0

4

13

6

4

5

0

0

0

0

0

0

0

0

0

0

0

0

0

0

303

63

0

0

0

0

0

0

0

0

0

0

0

0

0

0

0

0

0

63

1

1

1

0

0

0

0

0

29

4

4

25

25

2

2

2

7

0

0

3

3

4

4

0

0

0

24

22

22

2

2

240

0

0

0

0

0

0

0

240

129

129

129

1

1

1

7

7

7

3

3

3

100

100

100

145

140

140

0

0

0

140

119

119

0

14

1

12

0

1

7

7

0

0

0

0

0

0

0

0

0

0

0

0

0

0

0

0

0

0

0

0

0

0

0

0

0

0

0

0

0

0

0

0

0

0

0

0

0

0

0

0

0

0

0

0

0

0

0

0

0

0

0

0

0

0

0

0

0

0

0

0

0

0

0

0

0

0

0

0

0

0

0

0

0

0

0

0

0

0

0

0

0

0

0

0

5

0

0

0

0

0

0

0

0

0

0

0

0

0

0

0

0

0

0

0

5

5

5

5

0

0

0

0

0

0

0

0

0

0

0

0

0

0

0

1

1

1

1

1

1

0

0

0

0

0

0

0

0

0

0

0

0

0

0

0

0

0

0

0

0

0

0

0

0

0

0

172

45

45

45

1

1

17

17

9

0

9

18

9

9

0

0

0

0

0

127

127

127

13

13

0

103

0

0

39

1

63

7

7

4

2

2

610

83

83

0

0

0

83

83

83

0

0

0

0

0

0

0

0

0

0

0

0

0

0

0

0

527

124

112

89

17

63

9

23

3

18

2

0

12

12

11

1

0

0

0

0

0

0

0

0

0

393

3

3

3

0

0

0

0

0

0

0

0

0

0

0

0

0

0

0

0

0

0

0

0

0

390

390

7

383

10

10

0

0

0

0

1

1

9

2

7

0

0

0

0

0

0

0

12623

5343

0

0

0

0

76

2

2

2

74

2

2

0

1

1

0

1

1

70

6

64

0

0

0

0

94

94

54

0

54

37

37

3

3

187

0

0

0

187

4

4

2

2

0

0

181

181

0

0

0

44

44

1

0

1

0

43

1

0

16

26

0

0

0

0

0

0

0

331

331

0

0

15

3

0

0

8

4

0

0

0

0

0

0

2

2

0

0

0

0

0

0

36

2

0

3

19

1

2

9

0

0

70

3

54

9

4

0

0

0

188

2

1

20

0

0

2

0

0

0

1

7

0

10

16

0

0

31

3

24

0

18

0

35

6

9

0

1

2

0

0

20

8

2

0

10

0

0

0

0

0

0

0

0

0

1365

54

0

0

34

3

31

0

0

0

0

0

4

2

2

16

5

5

6

0

0

0

58

6

1

5

38

27

5

0

5

0

0

1

0

14

14

0

0

0

4

4

4

2

2

2

11

11

11

0

0

0

210

0

0

0

0

0

0

0

39

35

4

3

0

3

25

25

0

0

0

0

0

2

2

71

1

53

5

11

1

0

0

0

0

70

4

0

0

0

10

7

2

0

0

15

1

31

0

0

44

6

6

3

3

20

20

10

10

5

5

0

0

0

14

14

14

0

0

0

5

5

5

57

5

0

0

5

0

0

0

0

0

52

9

35

1

7

51

42

42

0

9

9

0

319

6

3

3

47

47

93

93

16

15

1

0

0

0

0

0

0

1

1

156

16

140

35

35

2

33

501

151

151

0

0

0

0

9

1

1

7

0

0

239

11

34

2

0

76

1

10

0

0

5

96

4

0

0

0

0

19

6

0

11

2

1

1

0

0

8

8

74

74

0

0

0

0

0

0

72

4

2

2

2

2

68

14

1

1

6

6

5

0

5

3

0

0

0

3

11

11

35

34

1

0

0

0

0

0

1

1

0

0

0

0

1

0

1

0

0

0

0

0

0

0

0

0

0

0

0

0

0

0

4

4

4

4

0

0

0

0

0

0

0

0

0

0

0

0

0

0

0

0

0

0

0

2391

2391

1

1

7

3

4

2225

2225

80

2

2

0

7

2

1

0

66

0

0

28

11

14

3

0

0

43

17

18

8

0

0

7

7

0

0

0

0

13

2

2

2

0

0

0

3

3

3

1

1

1

2

2

2

1

1

1

2

2

2

0

0

0

2

2

2

765

765

0

0

1

1

0

0

0

0

0

0

0

0

0

0

0

0

0

0

0

0

0

22

22

0

0

9

8

1

0

0

0

0

514

73

0

30

121

290

203

24

38

141

0

0

1

1

15

2

13

0

7280

0

0

0

0

0

0

0

0

0

0

0

0

11

11

11

11

0

0

0

2345

2

0

0

2

2

0

0

0

0

0

0

0

0

0

0

0

0

0

0

0

0

0

0

0

4

4

4

25

25

25

25

25

25

0

0

994

3

3

5

5

839

839

90

32

3

28

27

57

0

0

0

0

2

15

1

11

3

4

1

0

7

0

0

13

34

1

1

6

6

7

2

3

2

3

3

1

1

16

0

16

2

2

2

34

1

1

1

1

0

0

10

0

1

9

22

14

0

8

0

0

8

7

7

0

0

0

0

0

1

0

1

0

15

15

15

686

0

0

1

0

1

0

2

2

16

16

2

2

35

2

14

19

32

16

16

0

0

58

0

58

0

0

7

0

7

8

3

5

0

5

5

80

80

0

440

440

0

0

516

20

20

496

0

496

35

34

15

11

4

18

18

1

1

1

1

0

1

0

0

0

0

38

38

38

38

0

0

0

0

0

0

0

0

0

0

0

0

1

1

1

1

0

0

0

0

0

0

0

0

0

0

0

0

0

0

0

3153

1

1

1

3090

1

1

0

0

0

0

0

3069

3069

20

0

0

1

19

0

0

0

0

0

0

0

0

0

0

0

0

18

18

18

0

0

0

0

0

0

43

43

0

24

17

2

1

0

0

0

0

0

0

0

1

0

0

1

0

0

0

0

0

0

0

0

0

0

0

0

0

0

0

17

17

0

0

0

0

17

17

0

0

0

0

0

0

0

0

0

0

0

0

0

0

0

0

0

0

0

0

0

0

0

1592

4

2

2

2

2

0

0

0

0

0

0

0

0

0

0

0

0

475

365

365

31

31

27

27

52

52

59

59

59

0

1051

1051

1051

0

0

0

1

1

1

2

0

0

0

0

2

2

0

0

0

0

0

0

0

0

0

0

0

0

0

0

0

0

0

0

0

0

0

22

22

22

21

0

1

0

0

0

0

0

0

0

0

0

0

0

0

0

0

0

0

45

2

0

0

0

0

0

0

0

2

2

0

0

0

43

8

8

1

1

0

0

0

0

0

23

8

0

15

0

11

0

2

0

0

0

0

0

0

2

2

2

2

1

0

0

0

0

0

0

0

0

0

0

0

0

0

0

0

0

0

0

0

0

0

0

21

21

21

2

10

9

0

0

0

0

0

0

0

0

0

0

0

0

0

0

0

0

0

0

0

0

0

0

0

0

0

0

0

0

0

0

0

0

0

0

0

0

0

0

0

0

0

0

0

0

181

100

100

1

1

1

99

21

21

9

9

4

4

3

3

8

1

7

8

8

0

0

46

44

1

1

81

0

0

0

0

0

0

0

0

0

0

0

0

0

0

0

0

0

37

16

9

8

1

0

0

0

1

1

0

6

2

2

2

1

1

1

0

0

0

0

6

0

0

0

6

0

0

6

0

14

6

6

8

8

0

0

0

0

44

44

44

0

2

17

25

0

0

0

0

0

0

0

0

0

0

0

0

0

0

0

0

0

0

0

0

0

23

23

23

23

23

5

8

0

10

0

0

0

0

0

0

10894

0

0

0

0

0

10891

228

3

3

3

225

1

0

1

63

5

21

37

13

9

0

2

2

27

0

27

0

9

9

0

0

59

14

6

4

7

4

17

3

3

1

14

0

2

8

0

4

0

0

1

1

3

3

0

0

4

4

8

8

12

0

12

0

11

11

0

0

0

0

0

0

0

0

82

0

0

0

71

0

0

0

0

0

0

0

0

30

30

0

0

0

0

0

0

0

0

0

0

0

41

1

0

22

0

0

0

18

0

0

0

0

0

0

0

0

0

0

0

0

0

0

0

0

0

0

0

0

0

0

0

0

0

11

0

0

0

0

11

11

0

0

10446

0

0

0

0

0

0

0

0

0

0

0

3997

152

152

241

241

71

71

3533

3533

89

0

0

0

2

2

0

0

0

0

0

0

0

0

87

0

7

13

2

1

3

0

5

49

7

18

18

18

42

2

2

0

0

40

1

5

34

0

0

0

0

0

1

1

1

1788

1788

7

0

0

809

144

3

0

0

0

0

739

0

12

0

50

0

24

761

17

17

16

16

0

728

448

24

20

224

0

12

3697

0

0

0

180

0

180

2878

0

0

2878

0

0

549

549

0

0

0

0

12

0

0

12

0

0

0

0

0

0

0

0

0

0

0

0

0

0

78

0

0

13

0

0

0

0

0

0

0

56

3

0

6

0

0

0

0

0

0

0

0

53

53

0

53

0

0

0

0

0

0

0

0

0

0

7

0

0

0

7

5

0

2

3

2

2

0

0

0

0

0

0

0

0

0

0

0

0

0

0

0

128

25

0

0

3

3

2

2

20

6

12

2

11

0

0

11

0

6

5

0

0

0

0

28

1

1

0

0

15

3

12

6

6

6

6

0

0

0

0

12

4

4

8

8

0

0

0

0

52

0

0

43

1

5

7

20

6

4

6

3

3

0

0

1

1

0

2

0

0

0

0

0

1

0

0

0

0

0

0

1

0

0

0

0

0

1

1

1

1

1

0

0

2

2

2

2

2

0

0

0

0

0

0

0

0

0

0

0

0

0

0

0

0

0

0

63

49

0

0

0

0

0

0

0

0

0

0

0

0

0

0

0

0

0

0

0

0

0

0

0

0

0

0

0

49

48

48

48

1

1

1

0

0

0

0

14

14

14

8

4

4

3

3

3

1

2

0

0

0

0

1

1

1

1

1

1

0

0

5056

182

182

3

3

3

19

19

0

0

0

19

0

0

0

0

0

160

0

0

54

3

17

21

13

3

3

14

14

4

0

4

0

0

14

10

4

30

4

26

0

41

1

14

26

0

0

38

38

38

38

38

378

41

41

0

0

0

0

0

0

21

21

1

1

18

18

1

1

0

0

0

0

50

18

18

18

9

9

9

3

3

3

4

4

4

12

12

12

4

4

4

24

0

0

0

0

0

0

0

0

0

0

0

0

19

19

19

5

5

5

0

0

0

0

263

7

1

1

0

6

0

6

0

0

0

0

37

37

3

3

31

156

14

2

12

137

4

133

5

2

3

63

0

0

1

1

62

62

2132

1470

2

2

2

511

400

0

178

0

49

24

126

23

0

0

2

0

2

71

3

1

67

18

13

5

0

20

20

957

19

19

0

0

934

934

0

0

4

4

662

414

414

56

346

12

248

5

5

4

4

239

239

2249

1

1

1

1

0

0

0

0

0

0

0

0

0

0

0

0

0

0

0

0

46

46

1

1

0

0

7

1

6

5

5

1

1

0

0

10

5

1

1

3

9

1

5

3

8

8

0

0

0

0

5

5

1

1

0

0

0

0

0

0

1

1

77

29

29

3

26

0

0

0

0

33

33

8

8

7

10

15

12

0

12

0

0

3

3

0

0

188

188

0

0

0

188

12

77

65

0

2

10

0

22

0

0

130

40

2

2

32

32

6

6

4

1

1

3

3

8

8

4

4

4

4

4

8

8

1

7

66

23

23

20

20

23

23

12

1

1

0

1

0

0

11

0

0

11

11

0

204

204

21

21

0

0

183

13

16

0

0

0

143

9

0

0

0

0

0

2

10

10

10

10

0

0

0

10

10

2

2

8

2

0

5

0

1

0

0

377

299

49

49

2

2

120

120

0

0

0

0

0

0

0

2

0

2

27

0

27

0

99

99

78

12

1

3

8

0

0

44

0

0

7

1

3

0

0

8

0

0

0

0

5

0

4

2

0

0

2

1

3

3

5

16

16

0

0

0

6

6

96

96

0

0

0

0

2

1

1

0

0

6

6

85

2

3

6

9

43

22

0

0

0

0

0

1

1

1

1

1

1

1097

249

0

0

0

0

37

0

37

89

89

0

0

0

68

0

16

23

27

2

4

4

4

4

47

47

9

2

2

2

2

0

0

0

0

0

4

0

4

1

1

0

0

0

0

0

112

106

103

0

3

6

6

700

6

6

0

0

11

11

16

0

16

0

0

1

1

1

1

2

2

0

0

0

0

0

0

8

8

648

3

35

610

4

4

1

1

1

1

1

1

0

0

0

27

0

0

27

27

77

77

77

77

1

43

32

1

70

70

70

70

0

0

70

0

0

0

0

0

0

0

0

0

49

0

0

0

0

0

0

0

0

0

21

0

0

0

0

0

0

0

0

0

0

0

0

0

0

0

0

0

156

156

156

156

156

156

746

746

746

724

101

101

0

0

0

0

623

623

0

22

22

0

22

0

0

0

0

0

0

0

751

751

751

648

648

0

16

632

0

0

103

103

42

61

0

0

0

0

0

0

0

0

0

0

18

0

0

0

0

0

0

0

0

0

0

0

0

0

0

0

18

18

11

11

11

0

0

0

7

7

7

8

8

8

8

3

0

3

5

5

346

150

17

17

17

17

133

37

31

28

3

0

0

4

4

2

2

96

6

6

22

22

5

5

63

63

112

60

60

60

12

34

9

5

2

0

0

0

2

2

2

50

50

0

0

41

12

2

27

3

3

0

0

0

0

0

6

1

0

5

6

1

1

1

1

5

5

5

5

0

0

0

0

11

11

11

0

0

11

2

0

4

0

0

0

1

4

0

0

0

0

0

67

67

6

6

6

50

50

50

11

11

11

0

0

0

0

226

0

0

0

0

0

0

0

0

0

0

0

0

0

0

0

0

0

0

0

0

0

0

0

0

0

0

0

0

0

0

0

0

0

0

0

0

0

0

0

0

0

0

0

0

0

0

0

0

0

0

0

0

0

0

0

0

0

0

0

0

0

0

0

0

0

0

0

0

226

226

4

4

4

0

0

0

0

0

0

0

0

0

0

0

0

0

0

220

0

0

2

0

2

0

0

0

0

187

187

31

31

1

1

1

1

1

1

0

0

0

0

0

0

0

0

0

0

0

0

0

0

0

6

6

6

6

1

1

5

0

5

0

0

0

0

0

52

34

16

16

3

3

13

9

4

0

0

0

0

18

14

14

14

0

0

0

0

0

0

1

1

1

3

3

3

0

0

0

15

15

15

15

15

3

3

3

2

2

1

0

1

0

0

0

0

0

6

6

6

3

3

0

3

0

3

0

0

0

3

1

0

0

1

0

1

0

1

1

1

1

0

0

1

1

15010

8107

17

17

0

0

0

1

1

16

0

0

0

12

3

1

21

21

10

10

11

11

41

21

21

21

0

0

0

20

20

20

0

0

6

6

6

0

6

0

6579

6579

5

5

0

0

40

40

0

0

0

45

7

38

0

0

0

100

33

0

67

0

0

0

0

0

7

7

0

0

9

9

2

2

0

0

8

1

7

0

5

1

0

4

0

0

189

2

4

0

7

4

2

3

1

0

17

84

0

9

2

3

1

6

3

0

41

4

4

0

0

22

0

22

0

0

53

5

47

1

269

108

1

0

151

9

0

125

0

0

3

18

96

0

8

0

0

95

95

1286

17

0

2

1230

37

178

178

0

0

0

0

0

0

0

27

27

1

0

1

0

0

347

347

5

5

54

7

3

25

19

0

0

2

2

16

16

2

2

8

8

9

1

8

0

1

1

22

1

21

19

6

13

3585

9

303

0

33

0

142

2909

6

0

6

158

19

39

0

39

0

0

0

0

0

0

0

272

127

0

0

40

7

8

25

1

0

1

0

0

0

0

0

42

42

38

0

29

0

0

9

6

6

1

1

1

107

2

2

0

0

57

57

4

4

0

0

1

1

43

35

8

37

0

0

0

0

0

0

0

0

0

37

37

0

0

8

8

0

0

0

0

7

3

1

2

0

1

0

0

1

1

272

85

85

85

0

0

0

0

0

0

14

14

14

173

173

173

0

0

0

0

0

0

0

0

0

874

436

5

0

5

0

0

46

46

0

0

0

0

0

0

0

286

0

286

0

0

2

2

0

0

3

1

0

0

2

20

2

7

0

10

1

23

0

0

0

17

0

2

0

4

0

3

3

0

3

3

8

0

8

37

2

8

0

27

162

162

162

0

0

0

42

2

1

1

35

6

0

0

29

5

5

0

0

0

0

0

0

0

0

0

0

0

234

38

3

0

5

1

13

16

7

7

0

0

82

54

28

0

3

0

3

20

7

4

1

8

78

78

0

0

0

6

5

1

0

0

0

17

3

3

3

0

0

0

14

14

14

0

0

0

0

0

0

6808

8

8

8

8

749

197

9

9

7

7

4

4

32

25

0

6

1

0

74

4

70

15

15

55

55

1

1

0

0

552

0

0

0

0

0

0

0

552

18

0

0

0

1

8

0

1

0

469

15

1

17

0

0

15

7

0

0

0

0

2

2

0

0

0

2

2

0

0

3079

693

0

0

0

0

26

26

601

39

0

0

0

486

76

0

0

0

0

63

15

48

3

3

2386

26

26

89

89

267

236

31

11

0

11

1026

703

323

17

17

0

0

65

65

778

154

624

0

0

90

90

4

4

10

10

3

3

0

0

0

0

0

0

0

0

47

44

2

2

17

17

25

14

11

3

3

2

1

0

2

2

2

0

2

103

103

0

0

0

0

87

3

2

5

1

0

0

0

0

3

36

0

0

0

0

0

0

0

0

6

9

11

0

1

0

9

1

0

0

3

3

13

11

2

0

18

3

3

3

0

0

0

12

12

12

3

3

3

1998

1998

1998

1993

5

791

0

0

0

0

58

54

48

5

1

4

4

0

0

0

0

101

99

0

8

8

0

8

0

8

23

42

1

0

0

1

2

2

0

12

12

4

8

617

4

4

3

3

12

3

5

4

1

0

0

1

169

0

93

4

0

0

72

18

18

272

190

0

0

0

40

42

8

8

118

1

0

117

4

3

1

5

5

3

3

0

0

0

0

3

0

0

0

0

3

3

7

7

7

0

0

0

7

0

0

4

4

0

0

3

3

1

1

0

0

0

0

0

0

0

0

0

0

3

3

3

3

3

91

0

0

0

0

0

0

91

1

1

1

60

0

0

0

0

0

0

3

3

0

57

38

0

0

0

0

0

0

0

19

26

3

3

0

0

0

19

0

0

15

4

4

0

4

4

4

4

1

1

1

1

1

48

4

0

0

0

0

0

0

0

0

0

2

2

2

2

2

1

1

1

0

0

0

1

1

1

0

0

0

0

0

0

0

0

0

0

0

0

0

0

0

0

25

3

3

3

3

9

9

5

5

0

4

1

3

0

0

1

1

1

1

0

0

0

8

8

8

8

0

0

0

0

4

4

4

4

0

0

0

0

0

0

19

19

19

19

19

108

108

108

108

108

9

99

7

7

7

7

7

5

2

0

0

0

0

0

0

75

75

75

9

9

9

13

13

13

53

53

53

0

0

0

0

0

0

0

0

0

0

0

0

0

0

0

0

2

0

0

0

0

0

0

0

0

0

1

1

1

1

1

1

0

0

1

1

1

1

1

1

0

0

0

0

0

0
